# Review of Genomic Drivers of Thyroid Cancer and Their Clinical Implications

**DOI:** 10.3390/genes17010036

**Published:** 2025-12-30

**Authors:** Sobrina Mohammed, Daniel Mettman, Axel Hugo Breier, Vaishali Patel, Mariana Garcia-Touza

**Affiliations:** 1 Department of Endocrinology, Kansas City Veterans Affairs Medical Center, 4801 Linwood Blvd, Kansas City, MO 64128, USA; 2 Department of Pathology, Kansas City Veterans Affairs Medical Center, 4801 Linwood Blvd, Kansas City, MO 64128, USA; 3 Department of Research, Kansas City Veterans Affairs Medical Center, 4801 Linwood Blvd, Kansas City, MO 64128, USA

**Keywords:** genomic drivers, thyroid carcinoma subtypes, molecular profiling, risk stratification, redifferentiation strategies, preoperative planning

## Abstract

Over the past several decades, rapid advances in molecular genomics have transformed our understanding of thyroid malignancies and are increasingly integrated into international clinical guidelines. Mutational profiles and epigenetic events are now recognized not only as diagnostic and prognostic tools but also as predictors of therapeutic response. Papillary, follicular, oncocytic, medullary, and anaplastic thyroid carcinomas harbor distinct early driver mutations, such as BRAFV600E, RAS, and fusion events (RET, NTRK, and ALK), that cooperate with secondary alterations (TERT promoter, TP53, PIK3CA, and CDKN2A/B loss) to drive dedifferentiation, metastasis, and therapeutic resistance. Insights from The Cancer Genome Atlas (TCGA) and transcriptomic scoring systems (e.g., BRAF–RAS score) now link genotype to tumor morphology, metastatic tropism, and radioactive iodine refractoriness. These molecular insights have been incorporated into updated risk stratification frameworks, preoperative surgical planning, and treatment algorithms, informing the selection of kinase inhibitors, redifferentiation strategies, and enrollment in genotype-directed clinical trials for radioiodine-refractory disease. This review synthesizes recent evidence connecting genomic alterations to clinical behavior and highlights their translation into evolving approaches for thyroid cancer management.

## 1. Background

The classification and treatment of thyroid malignancies have traditionally relied on histopathology, staging, and clinical features. However, the last decade has witnessed a paradigm shift toward molecular characterization. Comprehensive genomic profiling has revealed a stepwise model of tumorigenesis: early driver mutations define tumor lineage, while late cooperating events accelerate progression and dedifferentiation. These insights now inform clinical guidelines, such as the 2025 American Thyroid Association (ATA) recommendations for somatic genomic testing, molecularly guided surgery, and systemic therapy selection. In this review, we address the genomic drivers of thyroid cancer and their clinical implications, synthesizing recent discoveries that connect genetic alterations to tumor morphology, metastatic behavior, therapeutic resistance, and opportunities for precision oncology.

## 2. Early Discoveries

Thyroid cancer is the most common endocrine malignancy [1], spanning a spectrum from indolent differentiated carcinoma (DTC) to aggressive anaplastic thyroid carcinoma (ATC). Over the past several decades, advances in molecular genomics have profoundly reshaped our understanding of its biology and clinical management.

Early evidence of tumor clonality emerged in the late 1980s, when X-chromosome inactivation analyses adapted from studies of hematologic cancers [2] revealed that benign adenomas and thyroid carcinomas arise from a single transformed progenitor cell, overturning earlier notions of purely hyperplastic multinodular goiters.

In the early 1990s, NIH-3T3 cell focus assays identified RET rearrangements and NTRK1 fusions as oncogenic drivers of papillary thyroid carcinoma (PTC), marking a paradigm shift toward the recognition of thyroid tumors as genetically initiated neoplasms [3,4,5]. RAS mutations were subsequently discovered as alternative mitogen-activated protein kinase (MAPK) pathway activators [6,7], particularly in follicular-patterned tumors, while TP53 mutations emerged as molecular hallmarks of ATC [8,9,10]. Around the same time, the discovery that germline RET mutations cause multiple endocrine neoplasia type 2 (MEN2) [11] and familial medullary thyroid carcinoma (MTC) revolutionized clinical care through genetic screening and prophylactic thyroidectomy.

By the early 2000s, PAX8–PPARG fusion was linked to follicular thyroid carcinoma (FTC) and some follicular-variant PTCs [12,13], demonstrating mutual exclusivity with RAS mutations and revealing a biological bridge between these subtypes. The identification of BRAFV600E in 2003 as the predominant driver mutation in PTC highlighted a potent, feedback-resistant MAPK activation pattern and reshaped the molecular classification of aggressive thyroid tumors [14].

### 2.1. Progression, Dedifferentiation, and Advanced Tumors

In 2013, the discovery of TERT promoter mutations, especially in combination with BRAF or RAS mutations, established a robust marker of dedifferentiation, recurrence risk, and metastatic potential [15,16,17,18,19,20]. Alterations in the phosphatidylinositol 3-kinase (PI3K)–AKT (Protein Kinase B) signaling pathway, including changes in Phosphatase and TENsin homolog (PTEN), phosphatidylinositol-4,5-bisphosphate 3-kinase catalytic subunit alpha (PIK3CA), and AKT1, have further clarified the molecular mechanisms driving progression from well-differentiated thyroid carcinomas to poorly differentiated thyroid carcinoma (PDTC) and ATC [20,21,22]. Whole-genome and exome studies of ATC revealed additional events: MTOR, NF1/NF2, MLH1/3, MSH5/6, ERBB2, EIF1AX, and RBM10 loss-of-function (affecting alternative splicing) and widespread copy-number changes such as 1q and 20q gains and 13q and 22q losses [20]. Many of these mutations appear as subclonal events within PTC, supporting stepwise evolution toward more aggressive phenotypes.

The Cancer Genome Atlas (TCGA) study of 2014 confirmed the low mutational burden of PTC, expanded the catalog of oncogenic fusions (EIF1AX, CHEK2, and PPM1D) [23], and led to the introduction of the BRAF–RAS score (BRS), a transcriptional signature that links MAPK signaling output to histologic phenotype, metastatic tropism, and radioactive iodine (RAI) refractoriness [24]. More recently, transcriptomic and epigenetic profiling have refined molecular subtypes, while updated clinical guidelines such as the 2025 American Thyroid Association Management Guidelines for Adult Patients with Differentiated Thyroid Cancer now integrate genomic information into risk stratification and therapeutic selection. Table S1 summarizes the key molecular discoveries and clinical advances in thyroid cancer (2013–2025).

### 2.2. Limitations of TCGA and Transcriptomic Systems

The Cancer Genome Atlas provides foundational molecular classification, but it is important to note that there are significant limitations for real-world application. TCGA established the thyroid differentiation score (TDS) as a composite measure of functional differentiation and identified mutually exclusive MAPK pathway mutations associated with different morphological variants [25]. However, TCGA data derive from surgically resected specimens, not preoperative fine-needle aspirations, limiting applicability to diagnostic decision making [26]. Similarly, TCGA data only include well-differentiated PTCs and do not address the more advanced subtypes of TC—namely, PDTC and ATC [26]. Rare histologic subtypes, such as oncocytic and poorly differentiated carcinomas, can be under-represented. In addition, most TCGA samples are derived from primary tumors rather than metastatic or radioiodine-refractory disease, restricting insights into molecular evolution during progression and treatment resistance.

Another key limitation is that TCGA offers a static snapshot of tumor genomics, providing cross-sectional rather than longitudinal data, which cannot fully capture intratumoral heterogeneity or clonal evolution over time.

Transcriptomic scoring systems face challenges in diverse populations. Gene expression profiles are context-dependent, influenced by the tumor microenvironment, inflammation, and sampling variability, which can affect reproducibility. Furthermore, while these scores provide valuable classification, their predictive power for clinical outcomes such as radioiodine responsiveness, metastatic potential, or success in targeted therapy remains limited. Technical challenges, including the need for high-quality RNA and standardized sequencing platforms, introduce variability and increase cost, limiting widespread adoption. Finally, most transcriptomic systems are derived from research cohorts like TCGA and offer only a static snapshot of tumor biology, lacking longitudinal data to track clonal evolution during progression or treatment. For these reasons, transcriptomic scoring systems are best used as adjuncts to histopathology and mutational analysis rather than standalone decision-making tools.

### 2.3. Oncocytic (Hürthle Cell) Tumors

Oncocytic tumors, distinguished by cells rich in abnormal mitochondria, were once poorly understood. Early work revealed loss-of-function mtDNA mutations (primarily complex I) but few nuclear drivers. In 2012, genome near-haploidization, monosomy or uniparental disomy across most chromosomes, sparing chromosome 7, was discovered in oncocytic carcinomas [27]. Subsequent comprehensive sequencing (2018) confirmed three hallmarks: (i) recurrent homoplasmic mtDNA mutations; (ii) widespread copy-number loss with near-haploidization; and (iii) nuclear DNA alterations such as RAS, TERT, and TP53 mutations in a subset [28,29]. Near-haploidization was later identified in one-third of oncocytic adenomas, suggesting a precursor relationship and supporting their reclassification as a distinct entity separate from FTC [24].

### 2.4. NIFTP (Non-Invasive Follicular Thyroid Neoplasm with Papillary-like Nuclear Features)

NIFTP is a follicular-derived thyroid tumor category formally introduced in 2016 [30]. It was later endorsed by the ATA (2017 guidelines and incorporated into the 2017 WHO endocrine tumor classification as a lesion with very low malignant potential. Globally, NIFTP accounts for roughly 2–10% of follicular-patterned thyroid neoplasms, with lower rates reported in Asian cohorts compared to North America and Europe [31].

Diagnosis requires strict histologic criteria. Earlier definitions excluded micro-tumors (≤1 cm) and oncocytic variants [30], but subsequent evidence demonstrated behavior of tumors measuring ≤1 cm (micro-NIFTPs) or with oncocytic features (oncocytic NIFTPs) similar to that of classic NIFTP, so these forms are now included [31]. Importantly, updated criteria no longer allow any true papillae, as even minimal papillary structures have been linked to lymph node metastasis [31]. Careful evaluation of the tumor border is essential to rule out invasion. NIFTP can be multifocal and may coexist with other thyroid cancers.

Molecularly, NIFTP behaves as a clonal neoplasm, with genetic alterations detected in most cases. RAS mutations—especially NRAS [32,33]—are the most common but are not specific, as they also appear in FTC and infiltrative FVPTC. Less frequent abnormalities include PAX8-PPARγ and THADA fusions and occasional BRAF K601E mutations [33,34]. Preliminary work suggests certain microRNAs may help differentiate NIFTP from infiltrative follicular-variant PTC [35], though this requires further study.

Long-term outcomes demonstrate excellent prognosis. Across multiple series, including cohorts retrospectively reclassified as NIFTP recurrence or persistent disease is exceptionally rare, with follow-up extending over a decade [36]. Lymph node metastases occur in <5% of cases, and distant spread is exceedingly uncommon [31].

NIFTP and related tumors of uncertain malignant potential fall into a pathological category with much lower risk than even the lowest risk differentiated thyroid cancers. Routine completion thyroidectomy, lymph node dissection, or radioactive iodine therapy is not recommended [31]. The best strategy for postoperative surveillance remains uncertain [31].

### 2.5. Radiation-Associated Thyroid Cancer

Ionizing radiation is the only firmly established environmental risk factor for sporadic thyroid cancer. The genetic basis of radiation-induced thyroid cancer was not elucidated until the marked rise in cases among children and young adults exposed to radiation following the Chernobyl nuclear power plant accident in April 1986 [24,37]. Post-Chernobyl studies revealed a dose-dependent enrichment of RET/PTC1, RET/PTC3, NTRK1, and AKAP9–BRAF fusions but low frequencies of BRAF point mutations, implicating nonhomologous end-joining DNA repair. Large-scale genomic analyses of >400 post-Chernobyl cancers confirmed these patterns and demonstrated that transcriptomic and epigenomic profiles correlated with driver type rather than radiation dose, while younger age at exposure conferred higher fusion prevalence [38].

### 2.6. Familial Syndromes

Several inherited disorders outside of the MEN syndromes are now known to heighten susceptibility to nonmedullary thyroid cancer. One of the first such connections was reported in families with familial adenomatous polyposis (FAP)/Gardner syndrome long before its molecular basis was understood. The later discovery of germline APC mutations clarified this relationship [39]. Individuals with FAP tend to develop a characteristic thyroid tumor with a cribriform and morular architecture, currently termed cribriform–morular thyroid carcinoma.

A separate cluster of hereditary disorders that predispose to thyroid involvement falls under the PTEN hamartoma tumor syndrome spectrum, of which Cowden syndrome is the most recognized example [40]. These conditions are driven by germline alterations in PTEN, and individuals frequently develop thyroid enlargement; benign nodules; or, in some cases, thyroid carcinoma. An increased likelihood of thyroid cancer is also characteristic of Carney complex, resulting from mutations in PRKAR1A [41], and Werner syndrome, which is associated with defects in the WRN gene [42] that plays a central role in maintaining genomic stability.

Newer evidence shows that DICER1 syndrome also predisposes individuals to thyroid involvement. The syndrome is caused by loss-of-function alterations in the DICER1 gene, which normally helps process microRNAs. Individuals carrying these mutations can present with thyroid nodularity, and some eventually develop differentiated thyroid malignancies [43]. Table S2 summarizes some common genetic syndromes associated with thyroid cancer risk.

## 3. Sex Bias in Thyroid Cancer

Women experience higher thyroid cancer incidence but better prognosis, particularly before menopause [44]. However, male sex is not an independent prognostic factor for disease-specific survival or recurrence after multivariable analysis [31]. The 2025 American Thyroid Association guidelines do not include sex as a consideration in risk of recurrence prediction modeling due to a lack of independent association [31].

The mechanisms underlying sex differences involve sex hormones, genetic and epigenetic factors, and immune-system variations [44]. Epigenetic and genetic factors contribute to sex bias in thyroid cancer through X-chromosome-linked genes, sex-specific histone modifications, and differential hormonal regulation of oncogenic pathways.

### 3.1. X-Chromosome Genetic Mechanisms

X-linked genes show sex-specific expression patterns that influence thyroid cancer susceptibility. The FOXP3 gene (located on the X chromosome) demonstrates both genetic and epigenetic associations with papillary thyroid cancer predisposition specifically in females. The CA and AA genotypes and A allele of the FOXP3 rs3761548 variant are associated with PTC risk only in women, and differential methylation at the FOXP3 promoter was observed between female PTC patients and controls [45]. This sex-specific pattern may reduce FOXP3 expression in immune cells, potentially explaining higher female incidence.

### 3.2. Epigenetic Modifications

Histone lysine demethylases (KDMs) exhibit sex-specific expression and cancer-related alterations in subcellular localization. Normal thyroids display distinct molecular signatures, with 44 sex-biased genes upregulated in males and 28 in females [46]. KDM5D is expressed exclusively in males, while KDM5C and KDM6A show sex-specific patterns [46]. Critically, thyroid cancer development is associated with loss of nuclear KDM expression and aberrant cytoplasmic accumulation, suggesting that disrupted epigenetic regulation contributes to tumorigenesis in a sex-dependent manner [46].

More broadly, epigenetic developmental mechanisms including X-inactivation, genes escaping X-inactivation, and imprinting pattern sex differences in metabolism, immunity, and tumor suppressor functions throughout life [47].

### 3.3. Hormonal–Genetic Interactions

Estrogen directly interacts with oncogenic signaling pathways in a sex-specific manner. In mouse models with PTEN loss (activating the PI3K pathway), estrogen significantly increases thyrocyte proliferation in females, with 52% of female Pten-/- mice developing follicular adenomas versus only 12% of males [48]. Estrogen activates both genomic and non-genomic pathways linked to MAPK and PI3K signaling—the same pathways frequently mutated in thyroid cancer through BRAF, RAS, and RET alterations [49]. This hormonal modulation of already activated oncogenic pathways may explain why females have a higher incidence despite males having worse prognosis.

This area warrants sex-specific research to develop more individualized therapies for advanced disease [44].

## 4. Applying Genomic Insights to Patient Care

### 4.1. Preoperative Risk Stratification

Molecular testing of fine-needle aspiration (FNA) samples has become a valuable adjunct to improve diagnostic accuracy in indeterminate thyroid nodules. Genomic alterations identified through these tests provide prognostic information that helps anticipate tumor behavior and determine the appropriate extent of surgery.

Multiple studies suggest that the coexistence of TERT promoter mutations with BRAFV600E is linked to more aggressive thyroid cancer behavior, although the impact of TERT mutations in small differentiated tumors is still unclear [18,50]. BRAFV600E alone is frequently present in both low- and high-risk papillary thyroid cancers and, by itself, does not reliably predict poor outcomes [51]. Other alterations, such as TP53, PIK3CA, and AKT1 mutations, also correspond with more aggressive or less differentiated disease, particularly when they occur together with BRAFV600E [52,53]. In contrast, RAS-type mutations, PTEN, and PAX8/PPARγ fusions, are more often found in follicular-patterned tumors and can even appear in benign lesions, limiting their specificity [23].

Molecular classifiers have been developed to help interpret indeterminate FNA cytology and to estimate tumor behavior. Retrospective studies grouping tumors into low-, intermediate-, and high-risk molecular profiles show that higher-risk patterns correlate with larger tumors, nodal spread, vascular invasion, and shorter recurrence-free survival [54,55,56]. Low-risk groups typically include RAS-like alterations; intermediate-risk groups include BRAF-like changes; and high-risk groups contain changes such as TERT promoter, TP53, AKT1, and PIK3CA mutations. Gene expression profiles have been incorporated into molecular risk stratification systems that classify thyroid cancers into low-, intermediate-, and high-risk categories based on clinical outcomes.

Alterations traditionally categorized as low risk, such as RAS mutations, BRAFK601E, and PAX8–PPARG fusions, are associated with encapsulated follicular-patterned lesions that behave indolently. In carefully selected patients without other high-risk features, these findings may support a more conservative surgical approach, such as lobectomy. In contrast, intermediate-risk alterations, including BRAFV600E, NTRK3 fusions, and RET fusions, are more commonly associated with classic papillary morphology, extrathyroidal extension, and a higher likelihood of lymph node metastasis. For these tumors, individualized decision making is essential, balancing oncologic control with the potential risks of more extensive surgery. High-risk profiles characterized by TERT promoter mutations, TP53, AKT1, and PIK3CA are predictive of vascular invasion, nodal disease, recurrence, and a more aggressive clinical course. For these patients, total thyroidectomy with possible prophylactic central neck dissection may be warranted.

Some studies have examined whether these molecular risk groups help guide the extent of initial surgery [31]. In selected cohorts, intermediate-risk profiles were associated with higher recurrence rates than low-risk profiles, especially in tumors measuring 2–4 cm [31]. However, because many studies include mainly patients undergoing total thyroidectomy and often lack uniform preoperative imaging, it remains uncertain whether molecular results should routinely influence decisions between lobectomy and total thyroidectomy [31]. Although noteworthy, gene expression-based classifiers have further refined this framework by linking molecular signatures with clinical outcomes, paralleling the low-, intermediate-, and high-risk categories used in the American Thyroid Association (ATA) risk stratification system.

Evidence is also mixed regarding the value of molecular testing for patients being considered for active surveillance of small (T1a) papillary cancers, with no consistent data supporting its routine use [57,58].

At present, the most promising application of molecular testing may be in selected patients with cT2N0 disease, where the optimal surgical extent is not always clear. More prospective studies using consecutive, unselected patients and formal cost-effectiveness analyses are needed before molecular testing can be widely recommended for the shaping of initial therapy [31]. For very advanced, invasive tumors, molecular testing is best incorporated into clinical trials, including studies exploring neoadjuvant approaches.

Cost–benefit analyses and prospective trials will be critical to determine the utility of molecular testing in this setting.

### 4.2. Delineating Clinical Validation Levels

Strong clinical validation exists for specific molecular markers in advanced disease management, while preoperative risk stratification remains largely investigational. For radioactive iodine-refractory (RAIR) differentiated thyroid cancer, tissue-based biomarker testing to identify actionable alterations (NTRK1/3 fusions, RET fusions, and BRAF V600E mutations) is recommended, with moderate certainty evidence from the American Thyroid Association [31]. These markers directly guide FDA-approved targeted therapies, including selpercatinib for RET-specific disease and dabrafenib plus trametinib for BRAF V600E-mutated anaplastic thyroid cancer. In contrast, preoperative molecular testing to guide surgical extent in cT1-cT2 cN0 disease is not routinely recommended due to the low certainty of evidence [31]. Studies demonstrating associations between high-risk mutations (BRAF V600E plus TERT promoter) and outcomes are predominantly retrospective, include heterogeneous populations (66% cytologically indeterminate nodules), and lack prospective validation in consecutive patients [31]. High-risk mutations appear uncommon in smaller intrathyroidal cancers, limiting clinical utility [31].

For cytologically indeterminate nodules (Bethesda III/IV), molecular tests demonstrate high sensitivity (91–100%) and negative predictive values (90–100%), enabling surgical avoidance rates of 50–68% [59]. However, these “rule-out” tests are validated primarily for diagnostic purposes rather than prognostic stratification.

### 4.3. Germline Testing

Tumor sequencing performed for clinical purposes can occasionally identify variants suggestive of an inherited cancer predisposition syndrome. Because somatic sequencing platforms are optimized for detecting acquired mutations and not germline variants, confirmatory germline testing should be pursued in conjunction with genetic counseling when a potentially clinically relevant finding is detected [31]. This is especially important for alterations in genes such as RET, PTEN, or DICER1, where identification of a germline mutation would have implications not only for the patient’s care but also for family members at risk [24,31]. Careful evaluation of personal and family history should guide decisions about further testing to avoid missed diagnoses of syndromes such as MEN2 or PTEN hamartoma tumor syndrome.

## 5. Systemic Therapy for RAI-Refractory Differentiated Thyroid Cancer (RAIR DTC)

### 5.1. Baseline Steps

The initial management of RAI-refractory DTC involves confirming the diagnosis of RAI refractoriness, optimizing local control with surgery, stereotactic body radiotherapy, or ablation when appropriate, in addition to maintaining TSH suppression to minimize stimulation of tumor growth. NGS testing should be performed to identify actionable genomic alterations that can guide systemic therapy choices.

### 5.2. First-Line Therapy

For most patients without an actionable genomic target, lenvatinib is the preferred first-line systemic therapy, as it has demonstrated superior progression-free survival. Sorafenib remains a validated alternative when lenvatinib is not suitable. For patients with RET, NTRK, or ALK fusions, highly selective kinase inhibitors such as selpercatinib, pralsetinib, larotrectinib, or entrectinib are recommended because of their high response rates and favorable toxicity profiles [31]. The LIBRETTO-531 trial represents a landmark phase III study demonstrating the superiority of selective RET inhibition in medullary thyroid cancer [60]. Selpercatinib showed superior progression-free survival compared to cabozantinib or vandetanib (median not reached vs. 16.8 months; HR 0.28, *p* < 0.001), with 12-month PFS of 86.8% versus 65.7% [60]. Importantly, treatment failure-free survival accounting for discontinuation due to adverse events also favored selpercatinib (HR 0.25, *p* < 0.001), with dose reductions required in only 38.9% versus 77.3% and treatment discontinuation in 4.7% versus 26.8% [60]. BRAFV600E-positive tumors can be treated with BRAF inhibitors such as dabrafenib or vemurafenib, with or without the addition of an MEK inhibitor such as trametinib, to enhance efficacy and mitigate resistance [31]. It is important to note that the phase II randomized trial showed no superiority of the dabrafenib plus trametinib combination over dabrafenib monotherapy [61]. Objective response rates of modified RECIST were 42% with dabrafenib alone versus 48% with combination therapy (*p* = 0.67), suggesting single-agent BRAF inhibition may be sufficient in this setting [61].

### 5.3. Resistance Mechanisms

Resistance to selective RET inhibitors occurs through both RET-dependent and RET-independent mechanisms [62,63]. The RET Δ898-901 deletion case demonstrated primary resistance to vandetanib and secondary resistance to selpercatinib after 12 months [62]. Critically, comprehensive genomic profiling of progressing lesions revealed no additional RET mutations but complete genetic loss of CDKN2A, CDKN2B, and MTAP genes, indicating RET-independent bypass resistance [62].

In a larger cohort, bypass mechanisms represented 75% of resistance cases following selective RET inhibitors, including RAS gene mutations (50%), FGFR2 and ALK fusions, and MYC p.P44L mutations [63]. RET solvent-front and hinge-region mutations accounted for only 25% of resistance mechanisms [63]. Tumor samples showed progressive dedifferentiation, with the mean Ki-67 index increasing from 7% at initial thyroidectomy to 17% pre-RETi and 40% post-RETi (*p* = 0.037), with more aggressive poorly differentiated histology emerging in three of six patients

### 5.4. Second Line and Beyond

Cabozantinib is the preferred second-line therapy for patients who progress after VEGFR-targeting multikinase inhibitors [24,31]. At each stage of therapy, enrollment in clinical trials should be encouraged, particularly for patients with rare molecular targets or for those eligible for novel combination strategies that may improve long-term outcomes.

### 5.5. Managing Progression on Gene-Specific Therapy

When disease progression occurs during gene-specific therapy, surgery or biopsy of a progressing lesion should be performed whenever feasible to obtain material for repeat NGS testing [31]. This allows for identification of on-target resistance mutations such as RET kinase-domain alterations or bypass mechanisms, including MET amplification or KRAS mutations. In patients with oligoprogression, where only a few lesions are growing while other sites remain stable, local therapies such as SBRT may be used while continuing the effective systemic drug. For patients with widespread progression, switching to a next-generation inhibitor or another class of therapy, ideally within a clinical trial, may prolong disease control.

### 5.6. Immunotherapy and Redifferentiation

Immune checkpoint inhibitors have shown limited but occasionally meaningful activity in RAIR DTC and are best reserved for tumors with biomarkers such as high tumor mutational burden or microsatellite instability-high status. Clinical trials evaluating combinations of VEGFR inhibitors with ICIs have demonstrated early signs of improved efficacy, although these regimens have not yet become the standard of care [24].

Redifferentiation therapy using MAPK-pathway blockade to restore iodine avidity has emerged as a promising strategy for a subset of BRAF- or RAS-mutant tumors. Studies using selumetinib or dabrafenib ± trametinib have demonstrated that some patients regain sufficient RAI uptake to allow for effective treatment, achieving meaningful responses. However, redifferentiation should be considered selectively or within the context of a clinical trial and is not recommended for non-genotype-selected patients [31].

## 6. Systematic Comparison of Redifferentiation Strategies

Redifferentiation approaches vary substantially in patient selection criteria, iodine uptake restoration rates, and response durability. MEK inhibitors (selumetinib and trametinib) and BRAF/MEK combination therapy (dabrafenib plus trametinib) represent the primary strategies, targeting the MAPK pathway to restore sodium iodide symporter expression [64]. Patient selection criteria differ across trials but generally include RAIR disease with BRAF V600E or RAS mutations, measurable disease on imaging, and adequate organ function [65]. Some protocols require documented progression, while others use redifferentiation as first-line therapy [65]. Iodine uptake restoration rates vary widely: 100% with selumetinib (*n* = 5), 60% with trametinib in one study (*n* = 10), and 88% with trametinib in another (*n* = 25) [65]. The 6-month partial response rate was 80% with selumetinib but only 20–32% with trametinib monotherapy [65]. For dabrafenib plus trametinib in BRAF V600E-mutated disease, a second course showed minimal added benefit (53% decrease from study entry but no change from second-course baseline) [65].

Response durability remains poorly defined. Time to retreatment with kinase inhibitors has been proposed as a benefit marker, but criteria for initiating such treatment are subjective and depend on tumor burden, growth rate, symptoms, and patient preference, making this an imprecise endpoint. Table S3 summarizes the systematic comparison of redifferentiation strategies and outcomes in BRAF V600E-mutated RAIR disease studies

### Overdiagnosis and Overtreatment Controversy

Overdiagnosis of thyroid cancer, particularly papillary thyroid carcinoma, is substantial, with recent estimates suggesting 63.5% of PTC cases in men and 79.5% in women represent overdiagnosis [66]. Early-stage PTC shows even higher rates: 73.9% overdiagnosis in men and 85.2% in women [66].

This overdiagnosis stems primarily from the widespread use of sensitive imaging technologies detecting clinically insignificant tumors [66,67]. Management strategies have evolved to reduce overtreatment, including active surveillance for low-risk papillary microcarcinomas, less aggressive surgical approaches, and selective use of radioactive iodine [67]. The 2025 ATA guidelines reflect this paradigm shift toward personalized, risk-stratified care that balances treatment risks against disease progression risks.

## 7. Future Directions

The future of thyroid cancer management will be shaped by emerging technologies and translational research. Liquid biopsy for circulating tumor DNA promises to detect minimal residual disease and resistance mutations in real time, while single-cell sequencing and spatial transcriptomics will refine our understanding of intratumoral heterogeneity. Combination strategies that pair targeted therapy with immunotherapy or redifferentiation approaches may overcome therapeutic resistance and improve survival outcomes. Guideline bodies such as the American Thyroid Association (ATA) and the European Society for Medical Oncology (ESMO) are already integrating molecular insights into recommendations for surgical planning, systemic therapy selection, and surveillance strategies. Ongoing clinical trials will further define optimal sequencing of therapies, expand treatment options for rare molecular subtypes, and incorporate patient-reported outcomes to ensure that therapeutic advances translate into meaningful improvements in quality of life.

## 8. Conclusions

Genomic discoveries have not only deepened our understanding of thyroid tumor biology but are actively reshaping clinical decision making. Molecular testing now complements traditional staging systems, informing surgical extent, systemic therapy choices, and follow-up strategies while highlighting potential germline risk for patients and families. The emergence of targeted inhibitors, redifferentiation therapies, and selective use of immunotherapy has expanded treatment options for advanced disease, yet therapeutic resistance and variable response remain ongoing challenges. Looking ahead, innovations such as liquid biopsy, single-cell sequencing, and combination therapeutic approaches are poised to refine patient selection and overcome resistance. Continued clinical trials and guideline evolution will be critical to fully integrate these tools into precision care, ensuring optimal outcomes for diverse thyroid cancer subtypes.

## Data Availability

No new data were created or analyzed in this study. Data sharing is not applicable to this article.

## Supplementary Materials

The following supporting information can be downloaded at: https://www.mdpi.com/article/10.3390/genes17010036/s1, Table S1. Key Molecular Discoveries and Clinical Advances in Thyroid Cancer (2013–2025). Table S2. Summarizes key genetic syndromes associated with thyroid cancer risk. Table S3. Summary of systematic comparison of redifferentiation strategies and outcomes in BRAF V600E-mutated RAIR disease studies [1].
