# Supplementary material for: Review of Genomic Drivers of Thyroid Cancer and Their Clinical Implications
Source: Genes (Basel). 2025 Dec 30;17(1):36. doi: 10.3390/genes17010036 (PMC12840629; doi:10.3390/genes17010036)
Supplement: Supplementary file 1 [file genes-17-00036-s001.zip › genes-4033421-supplementary.pdf]

Table S1. Key Molecular Discoveries and Clinical Advances in Thyroid Cancer (2013–2025)

Year	Discovery / Advance	Key Genomic Alteration(s) / Tool	Clinical Implication(s)
2013	TERT promoter mutations recognized in aggressive thyroid cancers	TERT promoter (often with BRAF or RAS)	Improved risk stratification for recurrence/metastasis; marker of dedifferentiation
2014	TCGA integrated genomic characterization of PTC; transcriptional scoring	Expanded fusion catalog; BRAF–RAS score (BRS)	Links genotype to histology, metastatic patterns, and RAI refractoriness
2016	Reclassification of indolent encapsulated FVPTC → NIFTP	Predominantly RAS-like alterations; occasional BRAF K601E	Reduces overtreatment; supports conservative surgery and avoids RAI in appropriate cases
2016	Genomic/transcriptomic hallmarks of PDTC and ATC refined	TP53, TERT, PIK3CA, PTEN, copy-number changes, etc.	Highlights stepwise progression; prioritizes molecular testing in advanced disease
2018	Genomic landscape of oncocytic (Hürthle cell) carcinoma clarified	Near-haploidization; recurrent mtDNA mutations; subset with RAS/TERT/TP53	Supports oncocytic tumors as distinct biology; informs prognosis and trial stratification
2019–2021	Radiation-associated thyroid cancer: fusion-enriched genomics described (post-Chernobyl)	RET/PTC, NTRK, AKAP9–BRAF; low BRAF point mutations	Improves etiologic understanding; reinforces fusion testing in selected settings
2018–2024	Selective targeted therapy expands for actionable alterations	RET fusions (selpercatinib/pralsetinib); NTRK fusions (larotrectinib/entrectinib); BRAF V600E (dabrafenib±trametinib)	Genotype-directed systemic therapy for RAIR disease and ATC; higher response rates vs. nonselective TKIs

2020–2024	Redifferentiation strategies mature	MAPK pathway blockade (e.g., MEK or BRAF±MEK inhibitors)	May restore iodine avidity in selected BRAF/RAS-driven tumors; enables repeat RAI in responders
2025	ATA guideline integration of somatic genomic testing and precision oncology pathways	NGS panels; actionable fusion testing; risk-framework updates	Standardizes molecular-guided surgery, systemic therapy selection, and germline follow-up when indicated

Abbreviations: PTC, papillary thyroid carcinoma; FVPTC, follicular variant PTC; NIFTP, non-invasive follicular thyroid neoplasm with papillary-like nuclear features; PDTC, poorly differentiated thyroid carcinoma; ATC, anaplastic thyroid carcinoma; RAI, radioactive iodine; RAIR, radioactive iodine–refractory; TKI, tyrosine kinase inhibitor; TCGA, The Cancer Genome Atlas.

Table S2. Summarizes key genetic syndromes associated with thyroid cancer risk.

Genetic Syndrome	Associated Gene(s)	Type(s) of Thyroid Cancer	Other Clinical Features
Multiple Endocrine Neoplasia Type 2 (MEN2)	RET	Medullary thyroid carcinoma (MTC)	Pheochromocytoma, hyperparathyroidism
Familial Adenomatous Polyposis (FAP)	APC	Papillary thyroid carcinoma (cribriform-morular variant)	Colonic polyposis, colorectal cancer
Cowden Syndrome (PTEN Hamartoma Tumor Syndrome)	PTEN	Follicular and papillary thyroid carcinoma	Breast, endometrial cancers, mucocutaneous lesions
Carney Complex	PRKAR1A	Follicular and papillary thyroid carcinoma	Cardiac myxomas, skin pigmentation, endocrine tumors

Werner Syndrome	WRN	Follicular thyroid carcinoma	Premature aging, diabetes mellitus, atherosclerosis
DICER1 Syndrome	DICER1	Multinodular goiter, differentiated thyroid carcinoma	Pleuropulmonary blastoma, ovarian tumors

Abbreviations: MTC = Medullary Thyroid Carcinoma; FAP = Familial Adenomatous Polyposis

Table S3. Summary of systematic comparison of redifferentiation strategies and outcomes in BRAF V600E-mutated RAIR disease studies

Strategy	Patient Selection Criteria	Iodine Uptake Restoration Rate	Partial Response Rate (6 months)	Response Durability	References
Selumetinib (MEK inhibitor)	RAIR disease, BRAF V600E or RAS mutations, measurable disease, adequate organ function; some protocols require progression, others permit first-line therapy	100% (n=5)	80%	Poorly defined; time to retreatment proposed as benefit marker, but criteria are subjective	Wells SA et al. [1]
Trametinib (MEK inhibitor)	Similar criteria: RAIR disease, BRAF/RAS mutations, measurable disease, adequate organ function	60% (n=10) and 88% (n=25) in separate studies	20–32%	Poorly defined; subjective retreatment criteria	Wells SA et al.

Dabrafenib + Trametinib (BRAF/MEK combination)	BRAF V600E-mutated RAIR disease	Minimal added benefit with second course (53% decrease from study entry, no change from second course baseline)	Not specified	Poorly defined; subjective retreatment criteria	Wells SA et al.
